# Physicians’ Attitudes Toward Euthanasia in Türkiye: A Cross-Sectional Survey of Ethical Heterogeneity and Decision-Making Patterns

**DOI:** 10.3390/healthcare14111554

**Published:** 2026-06-02

**Authors:** Halit Canberk Aydogan, Hanım Gökçe Arslan, Hacer Yaşar Teke

**Affiliations:** 1 Department of Forensic Medicine, Ordu University Training and Research Hospital, Ordu 52200, Türkiye; hacer.hgulderen2004@gmail.com; 2 Şırnak Forensic Medicine Branch Directorate, Council of Forensic Medicine, Şırnak 73000, Türkiye; hanmarslan@gmail.com

**Keywords:** euthanasia, end-of-life decision-making, physician attitudes, ethical heterogeneity, cross-sectional survey

## Abstract

**Highlights:**

**What are the main findings?**

- Within the study sample, support for euthanasia was strongly associated with personal willingness to consider it.
- Ethical acceptance of euthanasia varied substantially across clinical contexts, especially between terminal and non-terminal scenarios.

**What are the implications of the main findings?**

- The findings suggest that attitudes toward euthanasia among physicians may reflect multidimensional and context-dependent ethical positioning rather than a uniform moral perspective.
- The observed variability across clinical scenarios highlights the importance of context-sensitive ethical evaluation in end-of-life decision-making discussions.

**Abstract:**

**Background**: Physicians play a central role in end-of-life decision-making, yet their attitudes toward euthanasia remain complex and context-dependent. This study aimed to examine physicians’ attitudes toward euthanasia in Türkiye, focusing on ethical heterogeneity and decision-making patterns associated with support for its legal permissibility. **Methods**: A cross-sectional, web-based survey was conducted among 250 actively practicing physicians recruited via convenience sampling through physician-oriented social media platforms between November and December 2024. The primary outcome, support for the legal permissibility of euthanasia (Yes/No/Undecided), was analyzed using multinomial logistic regression. Additional analyses included item-wise ordinal logistic regression, latent class analysis, exploratory factor analysis based on a polychoric correlation matrix, and Firth penalized logistic regression. **Results**: Agreement with the standard definition of euthanasia was 94.4%. Support for legal permissibility was 44.0%, opposition was 35.2%, and 20.8% were undecided. Physicians who would not personally consider euthanasia had lower relative risk ratios for supporting legalization (RRR = 0.02, 95% CI: 0.01–0.06), and those who were undecided also had lower relative risk ratios (RRR = 0.19, 95% CI: 0.06–0.57). Agreement with euthanasia was 53.2% for terminal conditions and 18.4% for general scenarios. Latent class analysis identified three classes with proportions of 52.4%, 20.3%, and 27.4%. Exploratory factor analysis yielded two factors with eigenvalues of 4.58 and 1.47. In Firth penalized logistic regression, the odds ratio for not personally considering euthanasia was 0.034 (95% CI: 0.011–0.104). **Conclusions**: In this sample of physicians in Türkiye, attitudes toward euthanasia were heterogeneous and multidimensional. A substantial undecided group and context-dependent differences across clinical scenarios were observed.

## 1. Introduction

Euthanasia remains a highly controversial and largely unresolved domain globally, situated at the intricate intersection of jurisprudence, clinical ethics, and end-of-life decision-making [1,2]. Within this continuous debate, physicians are not merely passive practitioners or service providers; rather, they serve as moral agents whose authority and judgments ultimately determine the realization of these life-ending decisions [3,4]. The profound responsibility vested in healthcare professionals underscores that navigating the boundaries of life and death is inextricably linked to their core professional integrity and ethical obligations [5].

Despite the critical role of medical professionals, the majority of existing literature has assessed physician attitudes using reductive categories, often relying on simplistic binary frameworks that fail to adequately capture the multidimensional nature of ethical decision-making. This methodological oversimplification has generated a gap in the field, limiting the systematic modeling of structural heterogeneity and complex ethical patterns. Such reductionist approaches are fundamentally flawed, as they obscure the nuanced contingencies and overlapping dimensions that shape clinical perspectives on euthanasia [6].

Against this theoretical backdrop, Türkiye presents a highly compelling context characterized by the intersection of strong religious references, stringent legal restrictions, and rapidly evolving health system dynamics [7]. In this environment, where euthanasia is strictly prohibited by penal law and culturally contested, end-of-life debates generate a profound and multi-layered ethical tension among healthcare providers. Rather than serving merely as a localized case study, this unique socio-cultural and legal matrix constitutes a meaningful, yet relatively under-investigated, model for understanding how deep-rooted traditional paradigms collide with contemporary medical dilemmas [2,7].

To address these gaps, the present study departs from simple categorical assessments by conceptualizing physician attitudes towards euthanasia within a comprehensive, multidimensional framework. By investigating the underlying patterns and intricate decision-making processes, this approach specifically focuses on the ethical heterogeneity that has been severely neglected in prior literature. This perspective facilitates a more robust elucidation of the conflicting moral and professional paradigms that govern end-of-life care [8,9,10].

Ultimately, the objective of this study is not merely to describe physician attitudes towards euthanasia, but to rigorously examine their determinants and structural characteristics through a multi-layered analytical lens. Specifically, the relationship between support for the legal acceptance of euthanasia and an array of individual, clinical, and professional factors is evaluated analytically and systematically. Through this comprehensive methodology, the study aims to provide a profound understanding of the complex variables driving ethical decision-making among physicians facing end-of-life dilemmas.

## 2. Methods

### 2.1. Study Design

This study was designed as a cross-sectional, web-based survey conducted between 1 November and 31 December 2024. The primary aim was to evaluate physicians’ understanding of euthanasia-related concepts, their attitudes toward ethically controversial end-of-life practices, and the factors associated with support for the legal permissibility of euthanasia in Türkiye.

### 2.2. Participants and Recruitment

Eligible participants were physicians actively engaged in clinical practice in Türkiye during the study period. Physicians who were not providing active clinical services at the time of the survey or who declined participation were excluded.

Participants were recruited using a convenience sampling strategy through physician-oriented social media platforms. The questionnaire was distributed as an open-access Google Forms link across professional channels restricted to physicians.

According to platform-level activity indicators available to the investigators at the time of data collection, the survey link was accessed by approximately 1200 users (N_1_). Among these, 420 individuals initiated the questionnaire (N_2_). A total of 287 respondents completed all questionnaire items (N_3_). After exclusion of 37 respondents (N_4_) who did not meet the predefined eligibility criteria (e.g., not actively providing clinical services at the time of the survey), the final analytic sample consisted of 250 physicians. The excluded respondents were not omitted because of incomplete questionnaires; rather, they were excluded because they did not satisfy the study eligibility criteria.

Because recruitment relied on open-link dissemination rather than individualized invitations, a conventional response rate based on total exposure could not be calculated. Given the non-probability convenience sampling strategy, the sample cannot be considered representative of all physicians in Türkiye. Therefore, the findings should be interpreted as reflecting the characteristics of the study sample rather than population-level estimates.

Participation was voluntary, anonymous, and uncompensated. Electronic informed consent was obtained before participation.

### 2.3. Duplicate Prevention and Data Integrity

The survey platform was configured to restrict multiple submissions from the same respondent, thereby reducing the likelihood of duplicate entries. All returned questionnaires were screened for completeness before analysis, and only fully completed questionnaires were included. No item-level missing data were present in the final analytic dataset.

### 2.4. Questionnaire Development

The questionnaire was developed following a targeted review of the international literature on euthanasia and medical end-of-life decisions, including prior attitude instruments, comparative physician surveys, and cross-national studies of end-of-life decision-making [6,11,12]. Item selection was guided by ethical domains repeatedly emphasized in this literature, including patient autonomy and physician authority, distinctions between euthanasia and other end-of-life decisions, moral and religious considerations, and concerns regarding vulnerability, misuse, and broader system-level or economic consequences [6,12,13,14].

The questionnaire was not directly adapted from a previously validated scale; rather, it was conceptually informed by these sources and designed as an exploratory empirical ethics instrument to capture multidimensional ethical positions rather than a single latent construct. Accordingly, it was not intended to function as a psychometric scale but to explore ethical attitudes across distinct domains. Although formal content validity indexing and pilot psychometric testing were not performed, item wording and structure were iteratively reviewed by the authors to improve conceptual clarity, consistency with the operational definition of euthanasia, and interpretive comprehensibility across different clinical backgrounds.

### 2.5. Questionnaire Structure

The questionnaire consisted of three sections: (1) sociodemographic and professional characteristics, (2) knowledge and general attitudes toward euthanasia; and (3) responses to ethically controversial statements regarding euthanasia. Because the controversial ethical statements were intended to reflect potentially multidimensional attitudes, item responses were analyzed individually rather than aggregated into a composite score.

### 2.6. Conceptual Standardization

To reduce interpretive variability, a standardized definition of euthanasia was incorporated into the questionnaire. Euthanasia was defined as the intentional ending of a competent patient’s life by a physician through the administration of lethal medication based on the patient’s explicit and voluntary request. This definition was embedded directly within the relevant questionnaire items in order to promote conceptual consistency across respondents.

### 2.7. Sample Size Considerations

Sample size adequacy was evaluated using a prevalence-based approach for cross-sectional studies. Assuming a conservative prevalence of 50%, a 95% confidence level, and a margin of error of 6%, the minimum required sample size was calculated as 267 participants. Although the final analytic sample included 250 eligible physicians, the observed sample size was considered acceptable for exploratory multivariable and latent structure analyses given the study’s non-probability sampling design and the absence of missing item-level data.

### 2.8. Bias Considerations

Given the open-link, volunteer-based recruitment strategy and the ethically sensitive nature of the study topic, selection bias and social desirability bias cannot be excluded. Physicians with stronger views or greater interest in end-of-life ethics may have been more likely to participate. Although anonymity and voluntary participation were intended to reduce response distortion, the results should be interpreted as reflecting attitudinal patterns within the study sample rather than nationally representative prevalence estimates.

### 2.9. Statistical Analysis

Continuous variables were summarized as mean ± standard deviation, median with interquartile range, and range, as appropriate. Categorical variables were presented as frequencies and percentages. Wilson score 95% confidence intervals were calculated for key proportions reported in the Supplementary Descriptive Tables.

#### 2.9.1. Primary Outcome Analysis

The primary outcome was the response to the item: “Should euthanasia be legally permitted for competent patients who explicitly and voluntarily request it?” This variable had three categories (Yes, No, and Undecided). To preserve the informational value of the undecided category, the outcome was analyzed using multinomial logistic regression with No as the reference category.

This item was selected as the primary outcome because it directly represented physicians’ overall normative position regarding the legal permissibility of euthanasia. In contrast, the remaining questionnaire items were designed to explore multidimensional ethical attitudes, value orientations, and response patterns related to euthanasia rather than to constitute a unidimensional psychometric composite measure. Accordingly, exploratory factor analysis and latent class analysis were used to characterize the underlying attitudinal structure and heterogeneity of ethical perspectives within the sample, rather than to derive a single summary scale for primary inferential modeling.

Candidate predictors included age, sex, religious affiliation, marital status, having children, field of practice, professional title, years in profession, chronic disease status, prior exposure to explicit euthanasia requests in clinical practice, and personal willingness to consider euthanasia for oneself. Marital status and having children were included as sociodemographic variables potentially related to personal life circumstances and end-of-life decision-making perspectives. Potential multicollinearity between these variables was assessed during model construction.

Because some professional title categories contained sparse cell counts, professional title was collapsed before multivariable modeling into four broader groups: academic staff, specialist physicians, resident physicians, and general practitioners. Results were reported as relative risk ratios (RRRs) with 95% confidence intervals.

#### 2.9.2. Sensitivity Analysis for the Primary Outcome

As a sensitivity analysis, the same primary outcome was also treated as ordinal (No < Undecided < Yes) and analyzed using ordered logistic regression. The proportional odds assumption was assessed by comparing coefficients obtained from separate binary logistic splits across outcome thresholds. This model was treated as a sensitivity analysis rather than the primary analytic model.

#### 2.9.3. Item-Wise Ordinal Regression Analyses

Each controversial ethical statement (T1–T11) was analyzed separately as an ordinal outcome using multivariable ordinal logistic regression. Response categories were treated as ordered (Agree, Undecided, Disagree), such that higher ordinal categories represented greater disagreement.

Because multiple parallel item-wise models were estimated, *p*-values were adjusted using the Benjamini–Hochberg false discovery rate (FDR) procedure. Both raw and FDR-adjusted *p*-values were retained for reporting.

#### 2.9.4. Latent Class Analysis

To explore whether physicians could be grouped according to distinct response patterns across the controversial ethical items, latent class analysis was performed using responses to T1–T11. Competing models with two to six latent classes were estimated. Model selection was based on the Bayesian Information Criterion, with lower values indicating better fit. Estimated class proportions and selected conditional response probabilities were used to describe the resulting latent classes.

#### 2.9.5. Exploratory Factor Analysis

Because the controversial ethical items were ordinal, exploratory factor analysis was conducted using a polychoric correlation matrix. Internal consistency of the extracted dimensions was additionally evaluated using ordinal alpha coefficients derived from the polychoric correlation matrix. Dimensional structure was examined using eigenvalues and an interpretable two-factor solution. Factor loadings were inspected to identify clustering patterns among items. Model fit was summarized using the standardized root mean square residual, and the correlation between the extracted factors was also reported.

#### 2.9.6. Penalized Regression Sensitivity Analysis

To evaluate the stability of the primary associations in the presence of potentially sparse cells and separation, Firth penalized logistic regression was conducted as an additional sensitivity analysis. For this analysis, the primary outcome was dichotomized as Yes vs. No, and respondents with Undecided responses were excluded. Penalized odds ratios with 95% confidence intervals were reported.

#### 2.9.7. Statistical Software

Descriptive analyses and regression models were performed using IBM SPSS Statistics version 22.0 (IBM Corp., Armonk, NY, USA). Latent class analysis, exploratory factor analysis based on a polychoric correlation matrix, and penalized regression analyses were conducted using R software version 4.3 (R Foundation for Statistical Computing, Vienna, Austria) for advanced analyses.

A two-sided *p*-value < 0.05 was considered statistically significant unless otherwise specified.

### 2.10. Ethical Approval

The study protocol was approved by a Non-Interventional Scientific Research Ethics Committee (Approval Date: 11 October 2024; Approval No.: 2024/132). Participation was voluntary, electronic informed consent was obtained before survey initiation, and no personally identifiable information was collected.

## 3. Results

### 3.1. Sociodemographic and Professional Characteristics

A total of 250 physicians were included in the study. The cohort was relatively young (mean age: 36.92 ± 10.51 years). Female participants constituted a slight majority (56.0%). The sample was predominantly composed of physicians from internal medicine disciplines (71.2%), and most participants reported no chronic disease (81.2%). In terms of professional hierarchy, residents (38.4%) and specialist physicians (31.2%) represented the largest groups. Detailed sociodemographic and professional characteristics are presented in Table 1.

### 3.2. Knowledge and General Attitudes Toward Euthanasia

Physicians demonstrated a high level of agreement with the standard definition of euthanasia (*n* = 236, 94.4%) (Supplementary Table S1). Agreement rates were also high for distinguishing euthanasia from withdrawal of life-sustaining treatment (*n* = 228, 91.2%) and for recognizing the requirement of an explicit voluntary request (*n* = 231, 92.4%). Agreement with the concept of dying with dignity was reported by 52.4% (*n* = 131) of participants.

Regarding legalization, 44.0% (*n* = 110) of participants supported the legal permissibility of euthanasia, whereas 35.2% (*n* = 88) opposed it and 20.8% (*n* = 52) were undecided. In addition, 30.4% (*n* = 76) of participants reported that they would personally consider euthanasia under certain circumstances, while 35.6% (*n* = 89) reported that they would not and 34.0% (*n* = 85) were undecided.

### 3.3. Ethical Attitudes Toward Euthanasia

Responses to ethical attitude items are presented in Supplementary Table S2. Agreement that euthanasia is a personal decision based on individual values was reported by 69.6% (*n* = 174) of participants. Agreement with legalization in terminally ill patients was 53.2% (*n* = 133), whereas agreement with euthanasia for competent patients in general was 18.4% (*n* = 46). Agreement that severe deterioration in quality of life may justify euthanasia was 66.8% (*n* = 167). Agreement that euthanasia contradicts moral, religious, or natural principles was reported by 30.0% (*n* = 75).

Agreement with statements related to potential misuse and professional risks was 44.0% (*n* = 110) and 46.8% (*n* = 117), respectively.

### 3.4. Multinomial Logistic Regression Analysis

Multinomial logistic regression analysis was performed for responses to the item “G5-Should euthanasia be legally permitted for competent patients who explicitly and voluntarily request it?” using No as the reference category (Table 2). In the comparison of Yes vs. No, participants who reported that they would not personally consider requesting euthanasia for themselves had lower relative risk ratios than those who would consider it (RRR = 0.02, 95% CI: 0.01–0.06, FDR-adjusted *p* = 3.45 × 10^−9^). Participants who were undecided about personally considering euthanasia also had lower relative risk ratios (RRR = 0.19, 95% CI: 0.06–0.57, FDR-adjusted *p* = 0.0250). No variable in the comparison of Undecided vs. No remained statistically significant after FDR correction.

### 3.5. Item-Wise Ordinal Logistic Regression Analyses

Item-wise multivariable ordinal logistic regression analyses for ethical statements on euthanasia are presented in Table 3. After FDR correction, the item “G7-Under certain circumstances, would you personally consider requesting euthanasia for yourself?” was associated with multiple statements, including those related to personal decision-making, legal permissibility, clinical context, and ethical considerations.

### 3.6. Latent Class Analysis

Latent class analysis was performed using the ethical attitude items (T1–T11). Among the evaluated models, the three-class solution yielded the lowest Bayesian Information Criterion (BIC = 5208.5), compared with the two-class (BIC = 5237.8), four-class (BIC = 5280.7), five-class (BIC = 5359.2), and six-class (BIC = 5446.0) solutions. The estimated proportions of participants in the three latent classes were 20.3%, 52.4%, and 27.4%.

The distribution of responses differed across latent classes. In one class (52.4%), the probability of agreement was 0.92 for the statement “Requesting euthanasia is a personal decision based on individual values” and 0.88 for “Euthanasia should be legally permitted for terminally ill patients who explicitly and voluntarily request it.” In the same class, the probability of disagreement was 0.64 for “Euthanasia should not be performed because future medical advances may provide alternative treatment options” and 0.72 for “Euthanasia contradicts moral, religious, or natural principles.” In another class (20.3%), the probability of disagreement was 0.82 for “Euthanasia should be legally permitted for terminally ill patients who explicitly and voluntarily request it” and 0.97 for “Euthanasia should be permitted for competent patients who explicitly and voluntarily request it,” while the probability of agreement was 0.78 for “Euthanasia should not be performed because future medical advances may provide alternative treatment options,” 0.82 for “Euthanasia contradicts moral, religious, or natural principles,” and 0.75 for “Euthanasia may increase the risk of negligence or abuse among healthcare professionals.” In the third class (27.4%), the probability of undecided responses was 0.73 for “Euthanasia should be legally permitted for terminally ill patients who explicitly and voluntarily request it,” 0.55 for “Euthanasia should not be performed because future medical advances may provide alternative treatment options,” and 0.43 for “Euthanasia contradicts moral, religious, or natural principles.” Complete conditional response probabilities for all ethical attitude items across latent classes are presented in Supplementary Table S3.

### 3.7. Exploratory Factor Analysis

Exploratory factor analysis based on a polychoric correlation matrix was conducted for the same items. The first three eigenvalues were 4.58, 1.47, and 0.99. In the two-factor solution, factor loadings were 0.70 for “Requesting euthanasia is a personal decision based on individual values”, 0.84 for “Euthanasia should be legally permitted for terminally ill patients who explicitly and voluntarily request it,” 0.68 for “A severe decline in quality of life may justify considering euthanasia in patients who explicitly request it,” 0.55 for “Euthanasia should be permitted for competent patients who explicitly and voluntarily request it,” and 0.68 for “Euthanasia may be considered in cases involving prolonged and burdensome treatment when requested by the patient.” Loadings for the second factor were 0.72 for “Euthanasia may be misused as a cost-reduction strategy in healthcare systems” and 0.40 for “Euthanasia may increase the risk of negligence or abuse among healthcare professionals.” Ordinal alpha coefficients indicated acceptable internal consistency for the extracted dimensions (Factor 1: α = 0.79; Factor 2: α = 0.69). The standardized root mean square residual was approximately 0.093, and the correlation between the two factors was −0.88.

### 3.8. Sensitivity Analysis

As a sensitivity analysis, Firth penalized logistic regression was performed for the binary outcome (support vs. opposition to legal permissibility of euthanasia), excluding undecided responses (*n* = 198), and the results are presented in Table 4.

## 4. Discussion

A key finding observed within this study sample was the strong influence of physicians’ personal positions on euthanasia. Physicians who reported that they would not personally consider euthanasia were substantially less likely to support its legal permissibility (RRR = 0.02, 95% CI: 0.01–0.06), while those who were undecided also showed reduced support (RRR = 0.19, 95% CI: 0.06–0.57). The presence of a considerable undecided group further indicates that attitudes toward euthanasia are not strictly polarized but instead reflect varying degrees of ethical uncertainty. Similar associations across multiple ethical statements further support the interpretation that physicians’ views are shaped not only by abstract ethical reasoning but also by their personal orientation toward the issue. This pattern suggests that physicians may implicitly use their own hypothetical preferences as a reference point when evaluating ethically complex end-of-life decisions, thereby anchoring professional judgments in personal moral frameworks. This is consistent with previous studies suggesting that end-of-life decision-making is influenced by physicians’ own attitudes and their perceived relation to such decisions [15,16,17]. The substantial proportion of undecided respondents also aligns with prior evidence indicating that ethical judgments in this domain often remain fluid rather than fixed [18,19,20,21,22,23]. Importantly, the magnitude of the observed associations (RRR = 0.02) indicates that personal stance is not merely one of several contributing factors but may function as a strong contributing factor in ethical positioning in this context. Within the Turkish context, these findings suggest that physicians’ attitudes are shaped by the interaction between personal ethical positioning and broader sociocultural influences [24]. These findings indicate that physicians’ attitudes toward euthanasia are not solely constructed through professional norms but are deeply embedded in personal moral reasoning, with direct implications for consistency and variability in clinical decision-making.

Another important finding was the discrepancy between high levels of conceptual knowledge and heterogeneous ethical attitudes toward euthanasia. Although most physicians correctly identified the standard definition (94.4%) and the requirement of an explicit voluntary request (92.4%), support for legal permissibility remained limited (44.0%), with a substantial proportion opposing (35.2%) or remaining undecided (20.8%). Agreement with the concept of dying with dignity was also moderate (52.4%), indicating variability in how end-of-life concepts are interpreted despite shared technical knowledge. These findings suggest that knowledge alone is insufficient to determine ethical acceptance in end-of-life decision-making. This discrepancy may indicate that ethical judgments in this domain are not primarily driven by cognitive understanding but rather by value-based filtering processes through which individuals interpret clinical information. This interpretation is consistent with previous studies showing that cognitive understanding and clinical exposure do not necessarily translate into support for euthanasia, particularly in contexts shaped by moral and professional considerations [25,26]. From a practical perspective, this finding suggests that increasing knowledge or education alone may not lead to changes in physicians’ ethical positions unless underlying value systems are also addressed. In the Turkish context, this discrepancy likely reflects the interaction between formal medical knowledge and culturally embedded value systems [16]. Within the present sample, these findings suggest that ethical positioning in end-of-life care may be more strongly shaped by value-driven processes than by knowledge alone.

A clear context-dependent pattern was observed in physicians’ responses across different clinical scenarios. Support for euthanasia was substantially higher in terminally ill patients (53.2%) than in competent patients in general (18.4%), indicating that ethical acceptance varies according to clinical context, particularly the presence of severe and irreversible illness. This finding suggests that physicians tend to adopt a conditional rather than absolute ethical framework when evaluating euthanasia. This pattern may reflect a pragmatic ethical approach in which the perceived legitimacy of euthanasia is closely tied to the severity of suffering and the absence of therapeutic alternatives. Previous studies have similarly shown that support for assisted dying increases in cases involving terminal illness or intractable suffering and decreases in non-terminal contexts [26,27]. From a clinical perspective, this conditionality suggests that physicians’ decisions are likely influenced by situational thresholds rather than fixed ethical rules, potentially leading to variability in practice across similar cases. In the Turkish context, this pattern may reflect a stronger emphasis on the alleviation of suffering within established clinical and cultural frameworks of end-of-life care [16]. This context sensitivity indicates that ethical acceptance of euthanasia is contingent upon how clinical reality is framed, rather than being governed by a uniform normative stance.

The findings also indicate the coexistence of multiple and potentially conflicting ethical orientations among physicians. While a majority supported autonomy-based reasoning (69.6%), a substantial proportion expressed moral or religious opposition (30.0%) and concerns regarding misuse and professional risks (44.0–46.8%). This pattern suggests that support for euthanasia does not reflect a single coherent ethical position but rather a state of moral ambivalence, in which autonomy-based considerations coexist with concerns about harm, professional responsibility, and institutional trust. This coexistence may reflect an underlying ethical tension in which physicians are required to navigate competing principles, rather than consistently adhering to a unified moral framework. Previous studies have similarly shown that physicians often balance respect for patient autonomy with the obligation to avoid harm, resulting in persistent ethical tension in end-of-life decision-making [28,29]. Such tensions may become particularly pronounced in settings where legal, cultural, and professional expectations are not fully aligned, thereby increasing the complexity of ethical judgment. Concerns related to potential misuse and boundary expansion, including the “slippery slope” argument, have also been widely reported in the literature [30,31,32,33,34,35,36]. In the Turkish context, these overlapping positions may reflect the interaction between clinical reasoning and culturally embedded moral frameworks. This pattern suggests that physicians’ attitudes toward euthanasia are not defined by fixed ethical commitments but instead emerge from dynamic negotiation between competing values within specific clinical and societal contexts.

Analysis of response patterns and item structure demonstrated that physicians’ attitudes toward euthanasia are heterogeneous rather than uniformly distributed. Latent class analysis identified three distinct groups: an autonomy-oriented group (52.4%), a consistently opposing group (20.3%), and a substantial undecided group (27.4%), indicating the presence of intermediate ethical positions. Exploratory factor analysis further supported a multidimensional structure, with autonomy- and permissibility-related items loading on one dimension and system-level concerns on another (eigenvalues: 4.58 and 1.47). Together, these findings suggest that attitudes toward euthanasia may reflect partially overlapping but identifiable ethical response patterns rather than a fully unidimensional structure. However, the high inverse correlation observed between the extracted factors indicates that these dimensions should be interpreted cautiously within the exploratory framework of the study. Notably, the identification of a distinct undecided group may suggest that ethical uncertainty represents a potentially meaningful attitudinal position rather than merely a transitional or residual category. Within the Turkish context, this heterogeneity may reflect variation in value systems and professional perspectives. This interpretation is consistent with previous research demonstrating that aggregate approaches may obscure clinically and ethically meaningful subgroup differences [37,38,39]. These findings underscore the need to move beyond binary classifications and adopt analytical approaches that can capture the layered and structured nature of ethical decision-making. In this regard, the observed response patterns within the study sample appeared to be organized across identifiable but partially overlapping ethical dimensions rather than reflecting purely random variation.

Sociodemographic variables showed limited associations with attitudes toward euthanasia. In sensitivity analysis, not having children was associated with a higher likelihood of supporting legal permissibility (OR = 4.83, 95% CI: 1.50–15.62), whereas most other variables were not significant after adjustment. These findings suggest that demographic characteristics play a secondary role compared with underlying ethical orientations. This pattern indicates that physicians’ attitudes are more strongly anchored in internal value systems than in observable sociodemographic attributes. This interpretation is consistent with previous studies indicating that value-based factors are more influential than sociodemographic variables in shaping physicians’ attitudes toward euthanasia [15,40,41,42,43]. In the Turkish context, the association with parental status may reflect differences in experiential perspectives related to care and responsibility. However, the overall limited contribution of sociodemographic variables suggests that predictive models of ethical attitudes should prioritize psychological and value-based dimensions over structural characteristics.

The robustness of the findings was supported by sensitivity analyses using alternative modeling strategies. After excluding undecided responses, the association between personal stance and support for legal permissibility remained statistically significant in the Firth penalized logistic regression model (OR = 0.034, 95% CI: 0.011–0.104), with consistent direction and magnitude across models. These results indicate that the primary associations were not driven by sparse data or model specification. This consistency across analytical approaches strengthens confidence that the observed relationships reflect stable underlying patterns rather than methodological artifacts. Similar methodological studies have shown that key predictors in end-of-life research remain stable across different analytical approaches [44,45,46,47,48,49]. Importantly, the persistence of the personal stance effect across models reinforces its central role as a defining determinant of physicians’ ethical positioning. This robustness supports the internal consistency of the findings across alternative modeling strategies, but it should not be interpreted as evidence of population-level generalizability.

## 5. Limitations

The findings of this study should be interpreted within the context of several methodological considerations. First, the cross-sectional design limits causal inference, as physicians’ attitudes toward euthanasia may evolve over time in response to changing social and legal contexts. Second, the use of voluntary, social media-based recruitment may affect representativeness, as individuals with stronger engagement in end-of-life issues may have been more likely to participate. Accordingly, the results reflect attitudinal patterns within the study sample rather than population-level estimates. In addition, the final sample size remained slightly below the theoretically calculated prevalence-based target, which may limit the precision of population-level proportion estimates.

Third, reliance on self-reported data may introduce social desirability effects, particularly given the ethical and religious sensitivity of the topic. In addition, the study design did not allow differentiation between stable ethical uncertainty and alternative response processes such as satisficing, social desirability avoidance, or item-level ambiguity. Moreover, the questionnaire did not undergo formal psychometric validation procedures prior to administration. In addition, embedding a standardized definition of euthanasia within the questionnaire may have introduced anchoring or priming effects on subsequent responses, particularly for items related to autonomy and legal permissibility. The analytical model did not incorporate potentially relevant factors such as formal ethics education, palliative care training, experience in terminal patient management, or more nuanced dimensions of moral and religious orientation.

Finally, the variability observed across ethical attitude items reflects the inherently multidimensional nature of euthanasia-related views. This underscores the need for future research employing more refined and context-sensitive measurement approaches to better capture the complexity of ethical decision-making in end-of-life care.

## 6. Conclusions

In this sample of physicians in Türkiye, attitudes toward euthanasia appeared to be strongly associated with personal ethical positioning rather than conceptual knowledge alone. Support for legalization was strongly linked to physicians’ own willingness to consider euthanasia, while latent class and factor analyses revealed a heterogeneous and multidimensional attitudinal structure. Ethical acceptance was not uniform but conditional, increasing in the context of terminal illness and suffering, and constrained by concerns regarding professional responsibility and systemic risk. Taken together, these findings indicate that physician perspectives on euthanasia cannot be reduced to simple binary positions, but instead reflect complex, context-dependent ethical reasoning embedded within sociocultural and institutional frameworks.

## Figures and Tables

**Table 1:** Sociodemographic and professional characteristics of physicians.

Variable	Category	*n* (%)/Summary
**Age (years)**	Mean ± SD	36.92 ± 10.51
	Median (IQR)	33.00 (29.00–41.75)
	Range	24–70
**Gender**	Female	140 (56.0)
	Male	110 (44.0)
**Religious affiliation**	Islam	213 (85.2)
	Other	37 (14.8)
**Marital status**	Married	169 (67.6)
	Single	81 (32.4)
**Having children**	Yes	130 (52.0)
	No	120 (48.0)
**Field of practice**	Internal Medicine disciplines	178 (71.2)
	Surgical Sciences	39 (15.6)
	Basic Medical Sciences	33 (13.2)
**Professional title**	Resident	96 (38.4)
	Specialist physician	78 (31.2)
	Associate professor	24 (9.6)
	General practitioner	22 (8.8)
	Professor	15 (6.0)
	Assistant professor	15 (6.0)
**Years in profession**	Mean ± SD	11.83 ± 10.70
	Median (IQR)	8.00 (4.00–16.00)
	Range	0–46
**Chronic disease**	Yes	47 (18.8)
	No	203 (81.2)

**Table 2:** Multinomial Logistic Regression Analysis for the Item “G5-Should euthanasia be legally permitted for competent patients who explicitly and voluntarily request it?”.

Comparison	Predictor	RRR	95% CI	*p*-Value	FDR-Adjusted *p*-Value
**Undecided vs. No**	Specialist physician (vs. academic staff)	6.63	1.83–24.01	0.0040	0.0639
	Single (vs. married)	3.69	1.30–10.48	0.014	0.114
	Other religion/belief system (vs. Islam)	7.09	1.09–46.02	0.040	0.214
**Yes vs. No**	Would not personally consider requesting euthanasia for oneself (vs. would consider)	0.02	0.01–0.06	2.15 × 10^−10^	3.45 × 10^−9^
	Undecided about personally considering euthanasia for oneself (vs. would consider)	0.19	0.06–0.57	0.0031	0.0250
	Specialist physician (vs. academic staff)	4.49	1.40–14.34	0.011	0.060
	Not having children (vs. having children)	3.40	1.05–10.98	0.041	0.163

**Table 3:** Item-wise Ordinal Logistic Regression Results Showing Associations Remaining Statistically Significant After FDR Correction.

Item	Statement	Predictor	OR	95% CI	*p*-Value	FDR-Adjusted *p*-Value
T1	Requesting euthanasia is a personal decision based on individual values.	Would not personally consider requesting euthanasia for oneself (vs. would consider)	12.08	4.25–34.34	2.94 × 10^−6^	5.60 × 10^−6^
T2	Euthanasia should be legally permitted for terminally ill patients who explicitly and voluntarily request it.	Would not personally consider requesting euthanasia for oneself (vs. would consider)	31.29	12.35–79.26	3.83 × 10^−13^	1.41 × 10^−12^
T3	Patients with confirmed brain death are medically and legally dead; therefore, withdrawal of life-sustaining treatment in such cases is not euthanasia.	Would not personally consider requesting euthanasia for oneself (vs. would consider)	6.95	3.08–15.69	3.06 × 10^−6^	5.60 × 10^−6^
T4	A severe decline in quality of life may justify considering euthanasia in patients who explicitly request it.	Would not personally consider requesting euthanasia for oneself (vs. would consider)	5.85	2.43–14.04	1.12 × 10^−4^	1.07 × 10^−4^
T6	Euthanasia should be permitted for competent patients who explicitly and voluntarily request it.	Would not personally consider requesting euthanasia for oneself (vs. would consider)	9.62	4.39–21.09	1.85 × 10^−8^	4.42 × 10^−8^
T9	Euthanasia may be considered in cases involving prolonged and burdensome treatment when requested by the patient.	Would not personally consider requesting euthanasia for oneself (vs. would consider)	4.71	2.23–9.96	7.93 × 10^−5^	7.93 × 10^−5^
T7	Euthanasia should not be performed because future medical advances may provide alternative treatment options.	Would not personally consider requesting euthanasia for oneself (vs. would consider)	0.05	0.02–0.12	1.00 × 10^−12^	1.41 × 10^−12^
T8	Euthanasia contradicts moral, religious, or natural principles.	Would not personally consider requesting euthanasia for oneself (vs. would consider)	0.02	0.01–0.04	5.75 × 10^−16^	5.75 × 10^−16^
T11	Euthanasia may increase the risk of negligence or abuse among healthcare professionals.	Would not personally consider requesting euthanasia for oneself (vs. would consider)	0.35	0.18–0.69	0.0020	0.0033

T5 (“A patient’s request for euthanasia reflects giving up on life”) and T10 (“Euthanasia may be misused as a cost-reduction strategy in healthcare systems”) were included in the ordinal regression analyses; however, no associations for these items remained statistically significant after false discovery rate correction and therefore they are not displayed in the table.

**Table 4:** Firth Penalized Logistic Regression Analysis for Support of Legal Permissibility of Euthanasia.

Predictor	OR	95% CI	*p*-Value
Would not personally consider requesting euthanasia for yourself (vs. would consider)	0.034	0.011–0.104	2.8 × 10^−9^
Undecided about personally considering euthanasia (vs. would consider)	0.271	0.095–0.776	0.015
Not having children (vs. having children)	4.83	1.50–15.62	0.008

## Data Availability

The data are not publicly available due to ethical considerations and the sensitive nature of the data. De-identified data may be made available from the corresponding author upon reasonable request.

## Supplementary Materials

The following supporting information can be downloaded at: https://www.mdpi.com/article/10.3390/healthcare14111554/s1, Table S1: Knowledge and general attitude items; Table S2: Distribution of Physicians’ Responses to Controversial Ethical Statements Regarding Euthanasia; Table S3: Conditional Response Probabilities for T1–T11 Ethical Attitude Items Across Latent Classes.
