# Supplementary material for: Physicians’ Attitudes Toward Euthanasia in Türkiye: A Cross-Sectional Survey of Ethical Heterogeneity and Decision-Making Patterns
Source: Healthcare (Basel). 2026 Jun 2;14(11):1554. doi: 10.3390/healthcare14111554 (PMC13257177; doi:10.3390/healthcare14111554)
Supplement: Supplementary file 1 [file healthcare-14-01554-s001.zip › healthcare-4280414-supplementary.pdf]

Supplementary Table S1: Knowledge and general attitude items

Code	Question	Response	n	%	Wilson 95% CI
G1	Do you agree with the following definition of euthanasia: the intentional ending of a competent patient's life by a physician through the administration of lethal medication, based on the patient's explicit and voluntary request?	Yes	236	94.4	90.8–96.6
		No	11	4.4	2.5–7.7
		Do not know	3	1.2	0.4–3.5
G2	Withholding or withdrawing life-sustaining treatment is a medical decision distinct from euthanasia.	Yes	228	91.2	87.0–94.1
		No	4	1.6	0.6–4.0
		Do not know	18	7.2	4.6–11.1
G3	Euthanasia requires an explicit and voluntary request from a competent patient.	Yes	231	92.4	88.4–95.1
		No	4	1.6	0.6–4.0
		Do not know	15	6.0	3.7–9.7
G4	The concept of dying with dignity includes adequate symptom control, respect for patient autonomy, and psychosocial support at the end of life.	Yes	131	52.4	46.2–58.5
		No	51	20.4	15.9–25.8
		Do not know	68	27.2	22.1–33.0
G5	Should euthanasia be legally permitted for competent patients who explicitly and voluntarily request it?	Yes	110	44.0	38.0–50.2
		No	88	35.2	29.5–41.3
		Undecided	52	20.8	16.2–26.3
G6	Have you ever encountered a competent patient who explicitly requested euthanasia in your clinical practice?	Yes	46	18.4	14.1–23.7
		No	204	81.6	76.3–85.9
G7	Under certain circumstances, would you personally consider requesting euthanasia for yourself?	Yes	76	30.4	25.0–36.4
		No	89	35.6	29.9–41.7
		Undecided	85	34.0	28.4–40.1

Supplementary Table S2: Distribution of Physicians' Responses to Controversial Ethical Statements Regarding Euthanasia

Code	Statement	Response	n	%	Wilson 95% CI
T1	Requesting euthanasia is a personal decision based on individual values.	Agree	174	69.6	63.6–75.0
		Undecided	39	15.6	11.6–20.6
		Disagree	37	14.8	10.9–19.7
T2	Euthanasia should be legally permitted for terminally ill patients who explicitly and voluntarily request it.	Agree	133	53.2	47.0–59.3
		Undecided	64	25.6	20.6–31.4
		Disagree	53	21.2	16.6–26.7
T3	Patients with confirmed brain death are medically and legally dead; therefore, withdrawal of life-sustaining treatment in such cases is not euthanasia.	Agree	148	59.2	53.0–65.1
		Undecided	65	26.0	21.0–31.8
		Disagree	37	14.8	10.9–19.7
T4	A severe decline in quality of life may justify considering euthanasia in patients who explicitly request it.	Agree	167	66.8	60.7–72.4
		Undecided	42	16.8	12.6–22.0
		Disagree	41	16.4	12.2–21.5
T5	A patient's request for euthanasia reflects giving up on life.	Agree	124	49.6	43.5–55.7
		Undecided	56	22.4	17.7–28.0
		Disagree	70	28.0	22.8–33.8
T6	Euthanasia should be permitted for competent patients who explicitly and voluntarily request it.	Agree	46	18.4	14.1–23.7
		Undecided	62	24.8	19.9–30.5
		Disagree	142	56.8	50.6–62.8
T7	Euthanasia should not be performed because future medical advances may provide alternative treatment options.	Agree	61	24.4	19.5–30.1
		Undecided	90	36.0	30.3–42.1
		Disagree	99	39.6	33.7–45.8
T8	Euthanasia contradicts moral, religious, or natural principles.	Agree	75	30.0	24.7–35.9
		Undecided	62	24.8	19.9–30.5
		Disagree	113	45.2	39.2–51.4

Code	Statement	Response	n	%	Wilson 95% CI
T9	Euthanasia may be considered in cases involving prolonged and burdensome treatment when requested by the patient.	Agree	53	21.2	16.6–26.7
		Undecided	58	23.2	18.5–28.7
		Disagree	139	55.6	49.4–61.6
T10	Euthanasia may be misused as a cost-reduction strategy in healthcare systems.	Agree	110	44.0	38.0–50.2
		Undecided	48	19.2	14.8–24.5
		Disagree	92	36.8	31.0–43.0
T11	Euthanasia may increase the risk of negligence or abuse among healthcare professionals.	Agree	117	46.8	40.7–53.0
		Undecided	56	22.4	17.7–28.0
		Disagree	77	30.8	25.4–36.8

Supplementary Table S3: Conditional Response Probabilities for T1–T11 Ethical Attitude Items Across Latent Classes

Item	Response	Class 1 (52.4%) Autonomy-oriented	Class 2 (20.3%) Opposing	Class 3 (27.4%) Undecided
T1	Agree	0.92	0.11	0.41
	Undecided	0.05	0.13	0.37
	Disagree	0.03	0.76	0.22
T2	Agree	0.88	0.07	0.19
	Undecided	0.08	0.11	0.73
	Disagree	0.04	0.82	0.08
T3	Agree	0.81	0.22	0.48
	Undecided	0.12	0.31	0.39
	Disagree	0.07	0.47	0.13
T4	Agree	0.86	0.10	0.29
	Undecided	0.09	0.21	0.44
	Disagree	0.05	0.69	0.27
T5	Agree	0.21	0.72	0.41
	Undecided	0.18	0.17	0.39
	Disagree	0.61	0.11	0.20
T6	Agree	0.71	0.03	0.11
	Undecided	0.19	0.00	0.61
	Disagree	0.10	0.97	0.28
T7	Agree	0.09	0.78	0.25
	Undecided	0.12	0.09	0.55
	Disagree	0.79	0.13	0.20
T8	Agree	0.06	0.82	0.31
	Undecided	0.10	0.08	0.43
	Disagree	0.84	0.10	0.26
T9	Agree	0.74	0.08	0.17
	Undecided	0.14	0.12	0.51
	Disagree	0.12	0.80	0.32

Item	Response	Class 1 (52.4%) Autonomy-oriented	Class 2 (20.3%) Opposing	Class 3 (27.4%) Undecided
T10	Agree	0.38	0.69	0.44
	Undecided	0.24	0.15	0.35
	Disagree	0.38	0.16	0.21
T11	Agree	0.21	0.75	0.42
	Undecided	0.19	0.10	0.38
	Disagree	0.60	0.15	0.20
